# Selective Elucidation of Living Microbial Communities in Fermented Grains of Chinese Baijiu: Development of a Technique Integrating Propidium Monoazide Probe Pretreatment and Amplicon Sequencing

**DOI:** 10.3390/foods13111782

**Published:** 2024-06-06

**Authors:** Tao Bo, Jiaojiao Zhang, Enxiang Zong, Na Lv, Baoqing Bai, Yukun Yang, Jinhua Zhang, Sanhong Fan

**Affiliations:** 1 Key Laboratory of Chemical Biology and Molecular Engineering of Ministry of Education, Institute of Biotechnology, Shanxi University, No. 63 Nanzhonghuan East Road, Taiyuan 030006, China; botao@sxu.edu.cn (T.B.); lvna1258@163.com (N.L.); 2 Xinghuacun Fenjiu Distillery Co., Ltd., Fenyang 032200, China; 3 Shanxi Key Laboratory of Biotechnology, Taiyuan 030006, China; 4 Xinghuacun College (Shanxi Institute of Brewing Technology and Industry), Shanxi University, No. 63 Nanzhonghuan East Road, Taiyuan 030006, China; 202123118045@email.sxu.edu.cn (J.Z.); enxiangzong@foxmail.com (E.Z.); baoqingbai@sxu.edu.cn (B.B.); yangyukun@sxu.edu.cn (Y.Y.); ever840605@sxu.edu.cn (J.Z.); 5 School of Life Science, Shanxi University, Taiyuan 030006, China; 6 Shanxi Key Laboratory for Research and Development of Regional Plants, No. 63 Nanzhonghuan East Road, Taiyuan 030031, China

**Keywords:** propidium monoazide, amplicon sequencing, active microorganisms, fermented grains, Chinese Baijiu

## Abstract

The fermentation process of Chinese Baijiu’s fermented grains involves the intricate succession and metabolism of microbial communities, collectively shaping the Baijiu’s quality. Understanding the composition and succession of these living microbial communities within fermented grains is crucial for comprehending fermentation and flavor formation mechanisms. However, conducting high-throughput analysis of living microbial communities within the complex microbial system of fermented grains poses significant challenges. Thus, this study addressed this challenge by devising a high-throughput analysis framework using light-flavor Baijiu as a model. This framework combined propidium monoazide (PMA) pretreatment technology with amplicon sequencing techniques. Optimal PMA treatment parameters, including a concentration of 50 μM and incubation in darkness for 5 min followed by an exposure incubation period of 5 min, were identified. Utilizing this protocol, viable microorganism biomass ranging from 8.71 × 10^6^ to 1.47 × 10^8^ copies/μL was successfully detected in fermented grain samples. Subsequent amplicon sequencing analysis revealed distinct microbial community structures between untreated and PMA-treated groups, with notable differences in relative abundance compositions, particularly in dominant species such as *Lactobacillus*, *Bacillus*, *Pediococcus*, *Saccharomycopsis*, *Issatchenkia* and *Pichia*, as identified by LEfSe analysis. The results of this study confirmed the efficacy of PMA-amplicon sequencing technology for analyzing living microbial communities in fermented grains and furnished a methodological framework for investigating living microbial communities in diverse traditional fermented foods. This technical framework holds considerable significance for advancing our understanding of the fermentation mechanisms intrinsic to traditional fermented foods.

## 1. Introduction

The longstanding culinary legacy of China spanning millennia has engendered a varied spectrum of fermented comestibles, such as pickle, wine, vinegar and various fermented dairy products. Fermented comestibles, resulting from microbial growth metabolism and enzymatic processes, emerge as integral constituents of the dietary regimen for individuals [1].

Traditional fermented foods undergo natural fermentation within exposed fermentative settings, wherein microorganisms are domesticated over an extended period within specified fermentation parameters (temperature, pH and nutrients, etc.), thereby establishing a steadfast core microbiota [2]. The core microbial community dominates the transformation of raw materials into substances, bestowing upon fermented food its distinctive flavor profile [3]. In the case of Chinese vinegar, primarily manufactured via liquid and solid-state open fermentation methods, the enriched microorganisms involved in the fermentation process undergo metabolic processes leading to the production of various flavor compounds [4]. During vinegar fermentation, functional microorganisms such as *Acetobacillus*, *Lactobacillus*, and *Bacillus* produce saccharifying enzymes, amylases, and organic acids, exerting influence on the acidity and overall quality of vinegar [5]. Paocai is crafted through spontaneous anaerobic or microaerobic fermentation, with its flavor genesis intricately tied to the indigenous *Lactobacillus* in the raw material and the subsequent fermentation facilitated by microorganisms, such as *Bacillus* and *Pichia*, which are enriched by the fermentation [6]. In the realm of traditional fermented foods, the brewing process of Baijiu is based on solid-state fermentation, an intricate process amalgamating saccharification with spontaneous fermentation. Within this milieu, the microbial community executes its metabolic roles through complex interactions [7]. For example, during the fermentation of light-flavor Baijiu, flavor substances such as ethyl lactate and ethyl decanoate are positively correlated with the fungal genera *Dipodascus*, *Saccharomyces*, *Alternaria* and *Cosmospora*. These microorganisms interact in the direct or indirect synthesis of the mentioned esters or their respective precursor substances [8]. In summary, the various flavor substances found in fermented foods arise through the activities of functional microorganisms in the fermentation system. Precise analysis of the diversity and structure of functional microorganisms during the brewing process significantly impacts the accuracy of comprehending the fermentation mechanism.

Only the living microbial community can fulfill a distinct ecological role [9]; thus, the perpetual succession of the living microbial community drives the food fermentation process. Currently, the prevailing method for analyzing microbial communities is still based on high-throughput sequencing at the DNA level. However, since the DNA of dead cells can persist in the environment for an extended duration [10], high-throughput sequencing fails to discern between DNA signals emanating from dead and viable microorganisms. This limitation culminates in outcomes that incline toward an overestimate of the abundance and diversity of species [11]. To address the aforementioned challenges, Hu [12] used metatranscriptome technology to analyze the living microbial community during the fermentation process of Baijiu, and the results exhibited notable disparities compared to high-throughput 16S rRNA and ITS gene sequencings, for instance, *Aspergillus* was solely identified in amplicon sequencing results, while *Amanita*, *Baudoinia* and *Naumovozyma* were exclusively identified in the metatranscriptome results. This indicates that the results derived from high-throughput sequencing at the DNA level may not comprehensively and impartially portray the structure of the living microbial community and its succession during the process of food fermentation. At present, the studies of the composition and functionality of viable microorganisms during the process of food fermentation rely on traditional cultivation and metatranscriptome technology. However, the traditional cultivation method is time-consuming, as is DNA/RNA-based sequencing; in addition, the methods are not effective at isolating and culturing the majority of microorganisms, with only 0.1–1.0% of the naturally occurring microorganisms being successfully cultured [13]. Compared with the traditional cultivation method, metatranscriptome technology has the capacity to scrutinize both culturable and non-culturable microorganisms in fermented food systems. Nevertheless, the intrinsic challenges lie in the instability of RNA and the intricate nature of the environment in fermented foods, characterized by the abundance of enzymes and various metabolites, rendering the extraction of high-quality RNA a formidable task [14]. Additionally, the elevated expenses associated with sequencing pose constraints on the broader utilization of macro-transcriptomic technologies [15]. Therefore, the high-throughput analysis of the living microbial community in traditional fermented foods remains a persistent challenge.

Propyl azide bromide (PMA), as a high-affinity photoreactive DNA crosslinker, exhibits the capability to selectively associate with the DNA found within deceased cells possessing membrane permeability. Leveraging this distinctive attribute, PMA are widely used for the detection of viable microorganisms. For example, Xu [16] proposed PMA-CAMP for real-time and visual detection of active *Salmonella* in milk. This innovative approach enables the discernment of viable bacteria in artificially contaminated milk samples with a detection threshold of 10^2^ CFU/mL. Yang [17] improved the PMA-qPCR methodology and successfully completed the quantitative assessment of five viable bacteria in fermented milk, including *Lactiplantibacillus plantarum*, *Streptococcus thermophilus* and *Lactobacillus helveticus*; this refined PMA-qPCR technique enabled the analysis completion within a mere 3 h timeframe. PMA has been successfully applied to the monitoring of living microbial communities in a variety of fields, including food, gut microbiology and the environment [18,19,20]. PMA is an ideal method for the analysis of a living microbial community due to its exceptional selectivity for the DNA of dead cells. This characteristic imparts to PMA an outstanding level of specificity and sensitivity in the discernment of nonviable cells.

In the realm of food science research, current studies focus on the combination of PMA with real-time quantitative PCR (qPCR) for the detection of viable target microorganisms [21,22,23]. In fact, the method of sample pretreatment employing PMA offers an efficacious interface for the subsequent analysis of the living microbial community structure of traditional fermented foods through amplicon sequencing. Presently, only the living microbial communities in the fermented grains of Chinese strong-flavored Baijiu have been elucidated through the aforementioned methodology [24]. However, the elucidation of how the parameters for PMA pretreatment samples were established, along with a detailed optimization scheme, remains unprovided. In light of this problem, the present study focused on the establishment of the optimization scheme and process of the PMA pretreatment samples by taking the fermented grains of light-flavor Baijiu as a case study. Employing a fusion of qPCR and amplicon sequencing techniques, it confirmed the efficacy and reliability of PMA pretreatment in analyzing the amplicon of the living microbial community within the fermented grains of light-flavor Baijiu. This study aimed to offer a framework for establishing a methodology to scrutinize the living microbial community structure in fermented foods. Therefore, we hope this study will be a significant contribution to deepening the understanding of the fermentation mechanisms in traditional fermented foods.

## 2. Materials and Methods

### 2.1. Sample Collection and Suspension Preparation of Fermented Grain Samples

Fermented grain samples were collected from a local liquor distillery in Xinghuacun Town, Fenyang City, Shanxi Province, China (111°48′45″ E, 37°18′21″ N). In the production of light-flavor Baijiu, a dual fermentation process is employed using the same batch of sorghum raw material. The fermented grains from the first fermentation are termed “Dacha”. After distillation of the fermented grains (Dacha), the remaining sorghum was cooled and reintroduced with a saccharification fermenting agent, known as “Daqu”, for the second round of fermentation. At this stage, the fermented grains are referred to as “Ercha”. The fermented grains were sampled from the two fermentation cycles of Dacha and Ercha, and 100 g of fermented grains were collected at different fermentation time points. Subsequently, 2.5 g of fresh samples were added to 10 mL of PBS buffer (1 M, pH = 7.4), supplemented with 5–8 glass beads (Sangon Biotech (Shanghai) Co., Ltd., Shanghai, China) in number. After 20–30 min of vortexing at a speed of 4000 rpm and oscillating, the upper layer of the liquid was extracted, resulting in the formation of a 25% (*w*/*v*) suspension of fermented grains.

### 2.2. Preparation of PMA Working Solution

A total of 1 mg of PMA (SB-P4036, share-bio, Shanghai, China) was added to 98 μL of sterile distilled deionized water (ddH_2_O) under light-avoidant conditions. It was dissolved and thoroughly mixed to obtain a 20 mM storage solution, which was stored at −20 °C while shielded from light. The PMA working solution was diluted to 2 mM.

### 2.3. Preparation of Positive Control Group

*Escherichia coli* (*E. coli*), preserved in glycerol at a final concentration of 30% in the −80 °C freezer, were streaked onto Luria-Bertani (LB) agar plates and then incubated at 37 °C for 24 h. Subsequently, individual colonies were selected, inoculated into liquid LB medium, and cultured at 37 °C with agitation of 180 rpm on a shaker until reaching the logarithmic growth phase (OD_600_ ≈ 1.0). A total of 1 mL of *E. coli* culture solution was extracted in the logarithmic phase into a 1.5 mL centrifuge tube, incubated in a 95 °C water bath for 30 min to kill the bacteria and immediately placed on ice for cooling. In total, 50 μL of the heat-killed *E. coli* cell suspension was taken, and the suspension was plated on LB solid medium. Three replicates were set up in parallel for each group. The plates were inverted and incubated at 37 °C for 48 h, and the growth of colonies was observed. The absence of colony growth indicated the successful creation of a heat-killed bacterial suspension [25].

The positive control consisted of a suspension of heat-killed *E. coli* cells and suspensions formed from grains fermented for 0, 3, 7, 11, 15, 21, 24, and 28 days. In total, 400 μL of the heat-killed bacterial suspension was taken and centrifuged at 10,000 rpm for 5 min. The sediment was retained and then dissolved in 1 mL of a 25% (*w*/*v*) suspension of fermented grains to form a positive control bacterial suspension [24].

### 2.4. Establishment of PMA Pre-Treatment Method for Fermented Grains

The effectiveness of PMA binding to dead microorganisms was influenced by factors such as the concentration of the PMA, the duration of dark incubation, and exposure time [26]. To obtain an optimal PMA pre-treatment protocol for the samples, optimization was carried out separately for these three crucial factors. After applying factors such as PMA concentration, dark incubation time, and exposure time, there was a significant decrease in bacterial biomass, indicating the optimal treatment conditions. All experiments were conducted in triplicate.

#### 2.4.1. Selection of Fermented Grain Samples for Optimization of the PMA Pre-Treatment Protocol

DNA was extracted from fermented grain samples collected at 0, 2, 4, 6, 10, 15 and 24 days. Subsequently, DNA was amplified by qPCR. The fermented grains harboring the highest microbial density, encompassing bacteria or fungi, were subsequently utilized for refining the PMA pretreatment methodology.

#### 2.4.2. Optimization of PMA Concentration

PMA working solution was introduced to 1 mL of a 25% (*w*/*v*) suspension of fermented grains, yielding final concentrations of 0, 50, 100, 150, 200 μM, accompanied by a blank control (the suspension of fermented grains devoid of PMA treatment). Following thorough mixing, the mixture was placed in darkness for incubation and subsequently exposed utilizing a photolysis device (PL-PMA, Beijing Princess Technology, Beijing, China). The mixture was centrifuged at 10,000 rpm for 10 min, retaining the sediment. Subsequently, the DNA of the mixture was extracted using the Cetyltrimethylammonium bromide (CTAB) protocol [27]. The concentration of extracted DNA was quantified using the NanoDrop 2000 (Thermo Fisher Scientific, Waltham, MA, USA), and the optimal working concentration of PMA was determined based on the total bacterial biomass obtained from the qPCR results.

#### 2.4.3. Optimization of Dark Incubation Time

In total, 1 mL of a 25% (*w*/*v*) suspension of fermented grains was mixed with a final concentration of 50 μM of PMA working solution. The aforementioned mixtures were incubated in darkness for 0, 5, 10, 15, and 20 min, and subsequently, a photolysis process was conducted inside a photolysis device with light wavelength ranging from 465 to 475 nm within a centrifuge tube (Thermo Fisher Scientific, Waltham, MA, USA) for 10 min, with agitation occurring every 5 min during exposure. After the aforementioned steps, the mixture was centrifuged at 10,000 rpm for 10 min, resulting in the retention of the sediment. The genomic DNA of the mixture was then extracted using the CTAB method. The optimization of dark incubation time was assessed based on the total bacterial biomass obtained from qPCR results.

#### 2.4.4. Optimization of Exposure Time

The samples were prepared following the method outlined in Section 2.4.3 with a PMA concentration of 50 μM. The mixtures were mixed, incubated in darkness for 5 min, and then exposed to the photolysis device for intervals of 0, 5, 10, 15 and 20 min. A blank control group was included, consisting of the suspension of fermented grains without exposure treatment. Subsequently, the mixture was centrifuged at 10,000 rpm for 10 min, resulting in the retention of the sediment. The genomic DNA of the mixture was extracted. The optimal exposure time was determined based on the total bacterial biomass obtained from qPCR results.

### 2.5. Bacterial Strains, Plasmids and Cultivation

The DNA extracted from fermented grain samples was used as a template for PCR amplification using primers specific to bacteria and fungi. The forward and reverse primers of the bacteria were P1 (5′-CCTACGGGAGGCAGCAG-3′) and P2 (5′-ATTACCGCGGCTGCTGG-3′), and the forward and reverse primers of the fungi were Y1 (5′-GCGGTAATTCCAGCTCCAATAG-3′) and Y2 (5′-GCCACAAGGACTCAAGGTTAG-3′) [28]. Subsequently, the PCR products were loaded on 1% agarose gel, the target bands of 196 and 151 bp were isolated and recovered from the gel using a gel recovery kit (cat# DP219-03; Tiangen Biotech, Beijing, China). For vector ligation, the reaction solution consisting of the pMD-19T vector (cat# 6013, Takara, Japan), DNA of fermented grains, ddH_2_O and Solution I (cat# 6013, Takara, Shiga, Japan) was mixed and incubated overnight at 16 °C. The ligation products were added to *E. coli* DH5α Competent Cells (Beijing Solarbio Science & Technology Co., Ltd., Beijing, China), followed by incubation on ice for 30 min, a 90 s incubation in a 42 °C water bath, and addition of 800 µL LB medium without antibiotics under aseptic conditions. After cultivation at 37 °C for 1 h, a bacterial suspension was plated onto a selection agar plate containing a concentration of 100 μg/mL of ampicillin (Amp). Subsequently, the plate was sealed and incubated overnight at 37 °C. Afterward, the colonies were selected, transferred to LB liquid medium supplemented with Amp, and cultured at 200 rpm at 37 °C for 12 h. The recombinant plasmid DNA was extracted using the TlANprep Mini Plasmid Kit (cat# DP103-03; Tiangen Biotech, Beijing, China), and the concentration and purity of the plasmid were determined. The integrity of the plasmid was verified by PCR amplification and 1% agarose gel electrophoresis [29,30].

### 2.6. Amplicon Sequencing

DNA was extracted from fermented grain samples using the CTAB method, the concentration and purity of the DNA were measured using the NanoDrop 2000, and the quality was assessed by 1% agarose gel electrophoresis. The DNA was stored at −80 °C. Universal primers 515F (5′-GTGCCAGCMGCCGCGGTAA-3′) and 806R (5′-GGACTACHVGGGGTWTCTAAT-3′) were employed for amplifying the V4 variable region of the bacterial 16S rRNA gene, while primer sets ITS1F (5′-CTTGGTCATTTAGAGGAAGTAA-3′) and ITS2R (5′-GCTGGTTCTTCATCGATGC-3′) were utilized to amplify the fungal internal transcribed spacer region (ITS), followed by PCR amplification [31].

The DNA was purified and analyzed by amplicon sequencing on the NovaSeq 6000 sequencing platform. According to the characteristics of the amplified 16S region, a small fragment library was constructed, and the library was subjected to paired-end sequencing on the Illumina HiSeq4000 sequencing platform (Novogene, Tianjin, China). The downstream data obtained from Illumina NovaSeq sequencing underwent sequence assembly, quality control, and chimera filtering to yield valid data suitable for subsequent analyses. Finally, the classify-sklearn in QIIME2 is used to classify amplicon sequence variation (ASVs) [32]. The classify-sklearn naive Bayes taxonomy classifier in the feature-classifier plugin was employed to compare and identify sequences against the 16S rRNA Greengenes (version 13.8) database and the fungal ITS UNITE (version 8.0) database [33].

### 2.7. qPCR and PCR Reaction System and Procedure

qPCR was performed using a CFX96 Real-Time PCR System (Bio-Rad Laboratories, Hercules, CA, USA) with a final reaction volume of 20 μL. The reaction system for qPCR consisted of 10 μL of 2 × SYBR qPCR Master Mix (Yeasen Biotechnology (Shanghai) Co., Ltd., Shanghai, China), 0.4 μL of forward primers, and 0.4 μL of reverse primers, 1 μL of DNA template, and 8.2 μL of ddH_2_O. Each reaction was set up in triplicates to ensure the robustness and reproducibility of the results. Plasmid DNA for a standard curve was extracted and diluted in a 10-fold gradient: 10^−1^, 10^−2^, 10^−3^, 10^−4^, 10^−5^. The qPCR amplification procedure consisted of an initial denaturation step at 94 °C for 10 s, followed by 40 cycles of denaturation at 94 °C for 5 s, annealing at 58 °C for 15 s, and extension at 72 °C for 15 s. A final extension step was performed at 72 °C for 5 min [34].

The PCR amplification program was set as follows: pre-denaturation: 95 °C for 3 min; 27 denaturation cycles: 95 °C for 30 s; annealing: 55 °C for 30 s; extension: 72 °C for 45 s; final extension: 72 °C for 5 min [35].

### 2.8. Statistical Analyses

Data were analyzed using SPSS 21.0, and Origin 2021 was used for graphing stacking histograms and bar charts. Differences among groups were estimated using Tukey’s test and Student’s *t*-test, where *p* < 0.05 was considered statistically significant. The use of different letters such as “a” and “b” indicates significant differences at the *p* < 0.05 level; “**” indicates extremely significant difference at the *p* < 0.01 level, “*” indicates significant difference at the *p* < 0.05 level, and “ns” indicates no significant difference between the two groups.

## 3. Results

### 3.1. Selection of Fermented Grains for Optimization

**Figure 1:**
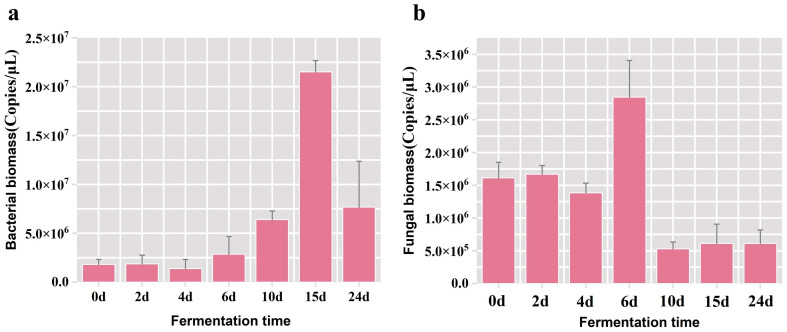
Variation in the bacterial and fungal biomass in fermented grains across various time points during the fermentation process. (**a**) The bacterial biomass in fermented grain samples; (**b**) the fungal biomass in fermented grain samples.

Fermented grain samples hosting the highest microbial population were meticulously chosen as the experimental subjects for refining the PMA pre-treatment protocol in the subsequent phase. Firstly, the fluctuation patterns of overall bacterial and fungal populations during the fermentation were analyzed. The results of qPCR showed a gradual elevation in the total bacterial biomass throughout the fermentation duration, peaking at 2.15 × 10^7^ copies/μL on the 15th day, followed by a subsequent decline (Figure 1a). The maximal of total fungal biomass was observed at 2.85 × 10^6^ copies/μL on the 6th day of fermentation (Figure 1b), and it was discerned that the fungal biomass in the fermented grains was one order of magnitude inferior to that of the bacterial population. Henceforth, in the subsequent experiments aimed at optimizing the pretreatment conditions of the PMA, we used samples of grains fermented for 15 days. The bacterial biomass served as the evaluative metric, ensuring the PMA dye was thoroughly bound to the DNA from dead cells in the fermented grain samples at any time point during the fermentation process.

### 3.2. Optimization of PMA Pre-Treatment Conditions for Fermented Grains

#### 3.2.1. Optimization of PMA Concentration 

**Figure 2:**
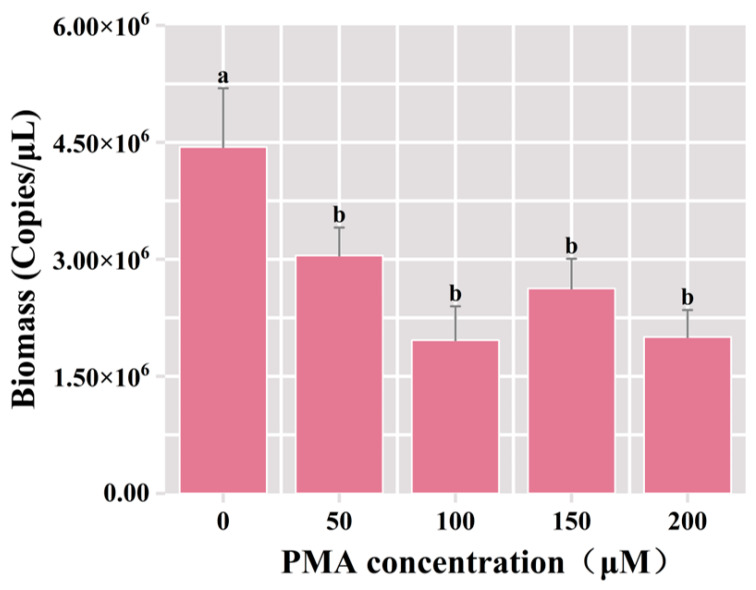
Variation of bacterial biomass in the samples of fermented grains under different concentrations of PMA treatment. “a” and “b” Superscript letters indicate significant statistical differences at *p* ≤ 0.05.

Following the incubation of samples with diverse concentrations of PMA, the DNA extraction ensued, and subsequent PCR amplification utilizing universal bacterial primers was conducted, followed by agarose gel electrophoresis. The results of qPCR showed that the total bacterial biomass was significantly reduced in the PMA-treated groups compared with the control group (*p* < 0.05). The total bacterial biomass decreased from 4.44 × 10^6^ copies/μL to 3.05 × 10^6^ copies/μL under the 50 μM PMA treatment, and the dead bacterial biomass accounted for 31.3% of the total bacterial biomass (Figure 2). In comparison to the group treated with 50 μM PMA dye, there was no significant difference in the number of viable bacteria in the PMA-treated groups employing the elevated concentration. This result indicated that 50 μM PMA effectively bound to the DNA of deceased cells in the fermented grain samples, proficiently differentiating viable microorganisms. Consequently, the optimal concentration for the PMA was determined to be 50 μM.

#### 3.2.2. Optimization of Duration for Incubation in Darkness 

**Figure 3:**
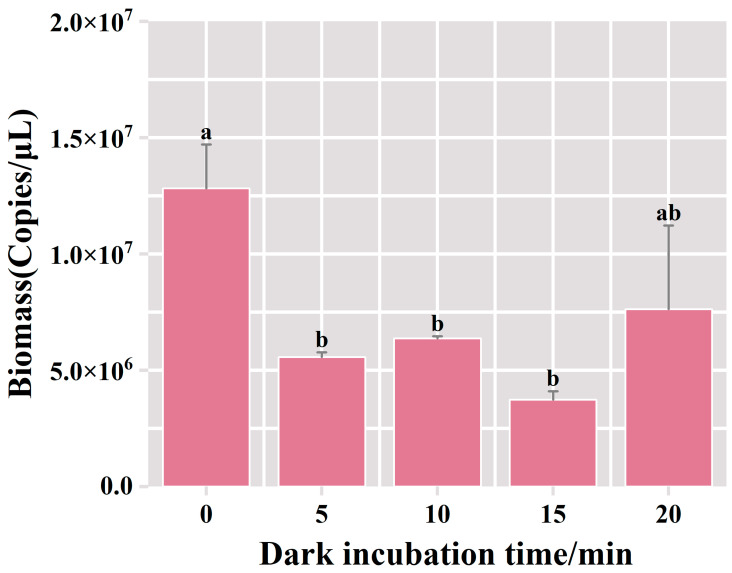
Variation of bacterial biomass in the samples of fermented grains under different dark incubation time treatments. “a” and “b” Superscript letters indicate significant statistical differences at *p* ≤ 0.05.

This section describes the results of 1% agarose gel electrophoresis, conducted on the DNA extracted from samples subjected to varying durations of dark incubation with the PMA dye. Within the 0 min dark incubation period in the PMA-treated group, the bacterial biomass resulted in 1.28 × 10^7^ copies/μL, surpassing the levels observed in the alternative experimental groups (Figure 3). In the PMA-treated group of dark incubation for 5 min, there was a noteworthy reduction in bacterial biomass, reaching 5.57 × 10^6^ copies/μL. This suggests that deceased bacteria comprised 56% of the overall bacterial content. There was no significant change in bacterial biomass with the increase in dark incubation time. This outcome suggested that the 5 min dark incubation treatment facilitated the complete binding of PMA to the DNA of deceased bacteria, effectively suppressing the amplification of dead bacterial DNA in the fermented grains. Therefore, the optimal duration for dark incubation was determined to be 5 min. 

#### 3.2.3. Optimization of Exposure Time

**Figure 4:**
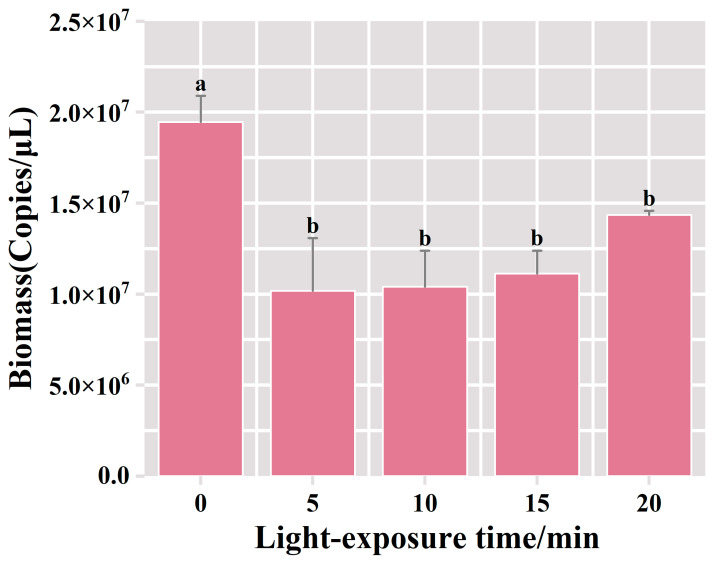
Variation of bacterial biomass in the samples of fermented grains under different exposure time treatments. “a” and “b” Superscript letters indicate significant statistical differences at *p* ≤ 0.05.

The qPCR results showed that the bacterial biomass reached 1.95 × 10^7^ copies/μL in the PMA-treated group without exposure. Compared with the unexposed control group, after 5 mins of exposure, the bacterial biomass in the PMA-treated group experienced a decrease to 1.02 × 10^7^ copies/μL, and deceased bacteria accounted for 48% of the total bacterial biomass in the suspension of fermented grains (Figure 4). No statistically significant difference was observed in the biomass of viable bacteria among the treatment groups with extended exposure durations. These results indicated that a 5 min exposure treatment was effective in fully binding PMA dye to the DNA of deceased bacteria, leading to the complete inhibition of dead bacterial DNA amplification. Furthermore, the prolongation of the exposure time did not result in a significant change in the detected viable bacterial biomass. Therefore, the optimal exposure time for this procedure appeared to be 5 min.

### 3.3. Assessment of the Binding Efficiency of PMA with the DNA of Deceased Bacteria in Fermented Grains under Optimal Treatment Conditions

**Figure 5:**
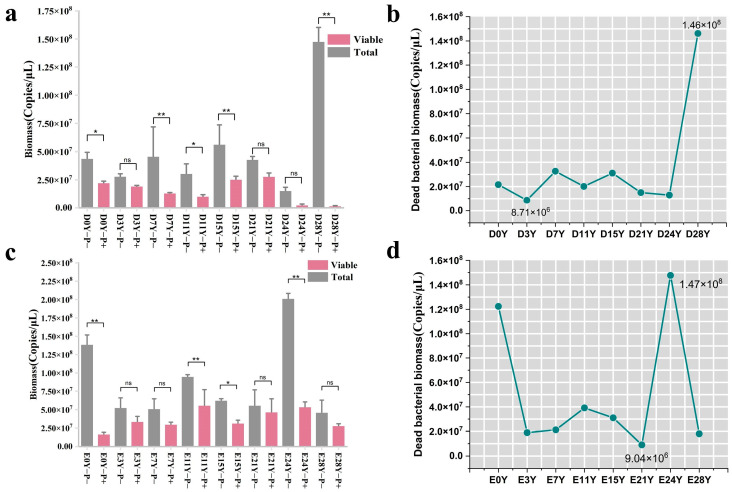
Effectiveness analysis of optimal PMA conditions for combining DNA from dead cells in fermented grains. (**a**,**c**) Comparison of changes of bacterial biomass before and after PMA treatment in the positive control group of fermented grains in Dacha and Ercha fermented grains; (**b**,**d**) curves of dead bacteria in the positive control group of fermented grains of Dacha and Ercha. “**” indicate significant statistical differences at *p* ≤ 0.01, and “*” indicate significant statistical differences at *p* ≤ 0.05. “ns” indicates no significant difference between the two groups.

In order to assess the binding efficiency of the PMA dye with the DNA of dead cells in fermented grains during the fermentation process, we used a mixture of heat-killed *E. coli* and suspensions of fermented grains obtained at different fermentation times as the positive control group. The upper threshold of PMA binding to the DNA of dead cells in fermented grains was determined via the positive control. Compared with the positive control group without PMA treatment, the total bacterial biomass of the positive control group, experiencing diverse fermentation durations, exhibited a decrease following treatment with the optimal PMA conditions (50 μM PMA, dark incubation for 5 min, exposure treatment for 5 min) (*p* < 0.01) (Figure 5a,c).

In the positive control group of Dacha fermented grains, the fluctuation range of dead bacterial biomass was detected using the PMA pretreatment method, amounting to 8.71 × 10^6^–1.46 × 10^8^ copies/μL, with dead bacteria constituting 31–99% of the total bacteria. In the positive control group of Ercha fermented grains, this range was found to be 9.04 × 10^6^–1.47 × 10^8^ copies/μL, with dead bacteria comprising 16% to 88% of the total bacteria (Figure 5b,d). The maximum total bacterial biomass in the fermented grain samples was 2.15 × 10^7^ copies/μL (Figure 1a). Nevertheless, the aforementioned findings suggested that, within the positive control cohort, subsequent to PMA treatment, the ascertained biomass of deceased bacteria surpassed the overall bacterial biomass observed in typical fermented grain samples. This implied that the refined pretreatment conditions facilitated proficient association of the PMA dye with the entirety of DNA derived from deceased cells in the fermented grain samples, thereby furnishing precise and dependable outcomes concerning the composition of the viable bacterial community. 

### 3.4. Evaluation of the Efficacy in Deciphering the Living Microbial Community Profile in Fermented Grains through the Coupling of PMA Pretreatment Technology with Amplicon Sequencing

#### 3.4.1. The α-Diversity of the Active Bacterial Community in Fermented Grains Exhibited a Markedly Reduced Level Compared to the Total Bacterial Community 

To investigate the feasibility of employing a PMA-coupled amplicon sequencing analysis method in the fermented grains, the 15-day fermented grains treated with PMA were chosen as the sequencing object. This experimental group, designated DV15, provided insight into the composition of the viable microbial community. In parallel, 15-day fermented grains without prior PMA treatment served as the sequencing control group (DT15), offering an overview of the entire microbial community structure. The rarefaction curves for bacterial and fungal communities attained saturation, implying that the quantity of sequenced data was judicious (Figure S3). Further data acquisition would not exert a substantial influence on the α-diversity index. 

**Figure 6:**
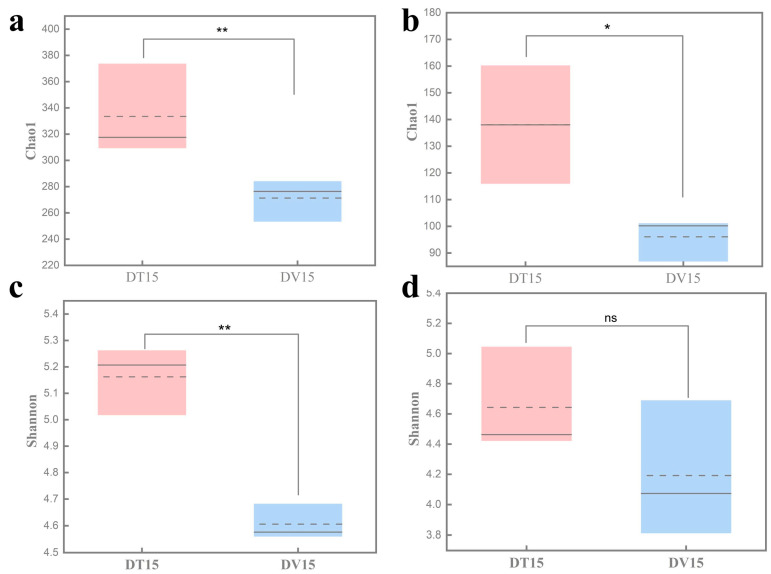
Comparative analysis of the α-diversity of total microbial community and viable microbial community in fermented grains. (**a**) Bacterial Chao1 index; (**b**) fungal Chao1 index; (**c**) bacterial Shannon index; (**d**) fungal Shannon index. “**” indicate significant statistical differences at *p* ≤ 0.01, and “*” indicate significant statistical differences at *p* ≤ 0.05. “ns” indicates no significant difference between the two groups.

The α-diversity serves as a comprehensive indicator of the structure of the microbial community, elucidating both the diversity and evenness within the sample’s microbial composition [36]. Compared with the untreated group DT15, the Chao1 and Shannon index of the bacterial community in the PMA-treated group DV15 was significantly reduced (*p* < 0.01), and no statistical difference was observed in Shannon indices of the fungal community between the DT15 group and DV15 group (*p* = 0.239). At the same time, we found that the Chao1 and Shannon index of the fungal community was lower than that of bacterial communities, indicating that the diversity of the viable bacterial community was reduced compared with the total microbial community, while the diversity of the viable fungal community was not significantly changed (Figure 6).

#### 3.4.2. Differences in Bacterial and Fungal Microbial Structure Composition of the DT and DV Groups

**Figure 7:**
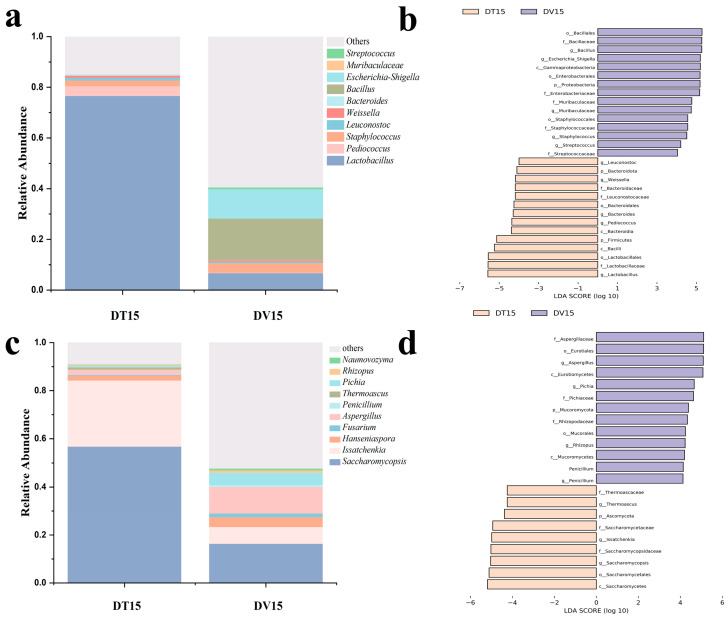
Composition and differences in the dominant microbial community at the genus level. (**a**,**c**) Top 10 bacterial and fungal community composition at the genus level in both experimental groups; (**b**,**d**) LEfSe identified the differentially abundant species of bacterial and fungal between the DT and DV group at the genus level.

Dominant genera were characterized by possessing an average abundance exceeding 1.0%. Within both experimental cohorts, *Lactobacillus* and *Staphylococcus* were discerned as predominant bacterial genera. *Pediococcus* and *Leuconostoc* were only identified as dominant bacterial genera in the DT15 group, while *Bacillus* (abundance accounted for 12.16% of viable bacterial) were only identified as dominant bacterial genera in the DV15 group. Compared to the DT15 group, the DV15 group exhibited a heightened relative abundance of *Bacillus*, *Staphylococcus* and *Streptococcus*, alongside a diminished relative abundance of *Lactobacillus*, *Streptococcus*, and *Bacteroidetes* (Figure 7a). It is noteworthy that the relative abundance of *Weissella* and *Leuconostoc* in the DT15 group was 1.31% and 0.75%, respectively; however, these two genera were not detected in the DV15 group.

In the fungal community, the dominant genera in both the DT15 and DV15 groups encompassed *Saccharomycopsis*, *Issacchenkia*, *Hanseniaspora* and *Aspergillus*. Compared to the DT15 group, the DV15 group exhibited an elevation in the relative abundance of *Hanseniaspora*, *Fusarium*, *Aspergillus*, *Penicillium*, *Thermoascus*, *Pichia* and *Rhizopus*, whereas the relative abundance of *Saccharomycopsis*, *Issachenkia* and *Thermoascus* experienced a decline. The relative abundance of *Fusarium* within the DV15 group diminished approximately 2.4-fold, from 56% to 23.05%. Concurrently, the relative abundance of *Issacchenkia* experienced a reduction of approximately 3.9-fold, descending from 27.4% to 7.04%. Meanwhile, *Thermoascus* was exclusively identified in the DT15 group (Figure 7c). These results indicated that the combination of PMA pretreatment and amplicon sequencing facilitated the monitoring and analysis of the changes in the living microbial community, and it was observed that disparities exist in the composition of the living microbial community compared to the total microbial community, whether considering the bacterial or fungal community.

The results of Linear discriminant analysis Effect Size (LEfSe) revealed distinctive bacterial genera, namely *Lactobacillus*, *Bacillus*, *Pediococcus* and *Staphylococcus*, distinguishing between the two experimental groups. Notably, the DT15 group exhibited elevated relative abundances of *Lactobacillus* and *Pediococcus* (Figure 7b). Regarding fungi, the discernible genera variance encompassed *Saccharomycopsis*, *Issatchenkia*, *Aspergillus* and *Pichia*. DV15 exhibited increased relative abundances of *Aspergillus* and *Pichia* (Figure 7d). 

#### 3.4.3. β-Diversity of the Bacterial and Fungal Community Revealed a Clear Separation between the DT and DV Groups

**Figure 8:**
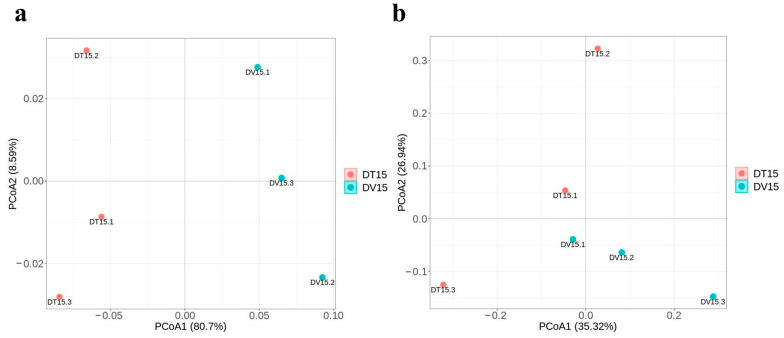
Comparative analysis of microbial β-diversity in fermented grains between groups DT15 and DV15. (**a**,**b**) PCoA of bacterial and fungal community composition based on DT15 and DV15 groups.

β-diversity was used to study the structural variation of microbial communities between samples and was visualized by Principal Coordinate Analysis (PCoA) [37]. Principal Coordinate Analysis (PCoA) was employed to evaluate the alterations in the structure of the living microbial community subsequent to PMA treatment in comparison to the overall microbial community structure within fermented grains. The results of PCoA showed distinctive clustering patterns in the microbial community structure between the DV15 and DT15 groups, discrepancies between the total microbial community structure and the living microbial community structure were evident (Figure 8a,b).

## 4. Discussion

In the course of Baijiu brewing, fermented grains, serving as the substrates for Baijiu distillation, harbor a diverse array of microorganisms. The microbial composition in fermented grains employed in Baijiu fermentation is notably intricate, mainly including bacteria, mold and yeast [38]. As high-throughput technology advances, the study of a Baijiu brewing mechanism from a microbial standpoint has gained increased depth, thereby profoundly enriching our comprehension of Baijiu fermentation principles. However, we observed a lacuna in the study of the fermentation mechanism of fermented grains, wherein microorganisms served as the subject of investigation without discrimination between deceased and viable microorganisms [24]. In fact, during the fermentation process of fermented grains, only the living microbial communities possess the capacity to incessantly metabolize nutrients, namely carbohydrates and proteins inherent in the raw materials. The metabolic activity results in the generation of diverse enzymes, alcohols, esters and flavor precursors, concurrently facilitating the degradation of some potentially detrimental substances present in the raw materials [39,40,41]. Therefore, the monitoring of the living microbial community stands as a means to complement and enhance the comprehension of the Baijiu fermentation mechanism.

PMA is a compound extensively employed in the surveillance of viable microorganisms [42]. At present, PMA combined with amplicon sequencing technology has been successfully applied to the analysis of the structure of living microbial communities in soil, sewage and human gut [20,43,44]. This study established the optimal pre-treatment protocol for PMA in microbes from fermented grain samples. The integration of PMA with amplicon sequencing was employed for the analysis of the living microbial communities, thereby confirming the viability and applicability of PMA in discerning living microorganisms within fermented grains. The pre-treatment parameters of the PMA dye exhibit variability across diverse sample types. For example, the optimal pre-treatment protocol for quantifying *Saccharomyces cerevisiae* and *Lactobacillus plantarum* during the brewing process of Hong Qu glutinous rice wine involved using a PMA concentration of 25 μM. The sample was incubated in the dark for 20 min, followed by an exposure treatment for 10 min. This protocol ensured that only viable microorganisms were quantified in the subsequent analysis [45]. In the studies of the microbial community structure in samples of strong-flavored fermented grains, the pre-treatment scheme for the samples involved a dark incubation step with a 50 μM PMA for 5 min, followed by an exposure treatment for 5 min [24]. The pretreatment conditions for fermented grain samples using PMA in the aforementioned studies were congruent with the findings of this study, thereby indirectly substantiating the validity of the results obtained in this study from an alternative perspective. Despite the utilization of distinct types of fermented grains in the two studies, these findings implied the potential applicability of this set of PMA pre-treatment parameters to diverse fermented grain samples. This result provided significant reference values for selecting PMA pre-treatment parameters in studies investigating the living microbial community structure of fermented grains in other flavored Baijiu. 

In this study, no discernible variance was observed in the total and viable fungal communities. However, it was determined that the diversity of living bacterial communities in the DV15 group was significantly reduced compared to the total bacterial communities in the DT15 group. These results implied the prevalence of deceased cells within bacterial communities, juxtaposed with fewer deceased cells within fungal communities. The reasons for these differences may stem from the binding of the PMA to the DNA of dead cells in the fermented grains, thereby influencing the structural composition of the microbial community, as depicted in the sequencing outcomes. Consequently, this change affected the relative abundance proportion of the microbial community [46]. Alternatively, dead cells may arise from the accumulation of dead bacterial entities generated during the initial phase of fermentation in the fermented grains. Furthermore, owing to the adaptive capacity of certain microorganisms to thrive within the fermentation milieu of fermented grains, their presence in the living microbial community was augmented by the relatively limited occurrence of deceased cells within the fermented grains [47]. 

In the sample of grains fermented for 15 days, the dominant microbial composition in both the total microorganisms and the viable microorganisms was similar, with disparities discernible solely in relative abundance. The dominant genera of viable bacteria in the fermented grains were mainly *Lactobacillus*, *Staphylococcus* and *Bacillus*. Meanwhile, the dominant genera of viable fungi identified were *Saccharomycopsis*, *Issatchenkia*, *Hanseniaspora* and *Aspergillus.* The study by Wang [48] employed macro-transcriptomics to investigate Luzhou-flavor fermented grains, revealing transcriptional activity in *Lactobacillus*, *Saccharomyces*, *Bacillus* and *Staphylococcus*, all of which occupied dominant positions. This finding aligned with the structure and composition of the predominant viable bacteria observed in the present study. These findings indicated the rationality of the sequencing outcomes of our investigation, further suggesting the significant involvement of the aforementioned living microorganisms in shaping the diverse flavor profiles of Baijiu.

The main differential bacterial genera between total and viable microorganisms were *Lactobacillus*, *Bacillus* and *Pediococcus*. During the fermentation process, *Lactobacillus* and *Pediococcus* stood out as the predominant lactic acid bacteria inhabiting the fermented grains. Their metabolic activities were notably influenced by factors within the fermentation milieu, such as oxygen availability and ethanol concentration. Meanwhile, they enzymatically generate lactic acid, thereby reducing the pH of the fermentation milieu and imposing acid stress on other microorganisms [49]. In our study, we observed a higher relative abundance of *Lactobacillus* and *Pediococcus* in the DT15 group compared to the DV15 group. This disparity may be attributed to the ethanol stress exerted by *Saccharomyces cerevisiae*, which produced alcohols within the identical fermentation milieu. This ethanol stress adversely impacts the metabolic processes of lactic acid bacteria, consequently hindering the growth of both *Lactobacillus* and *Pediococcus* [50]. *Bacillus* can utilize enzymes to efficiently hydrolyze starch into reducing sugars. This crucial process provides a nutrient-rich substrate that facilitates subsequent fermentation processes [51]. The relative abundance of *Bacillus* in the DV15 group surpassed that of the DT15 group, potentially attributed to *Bacillus* possessing traits of thermal and acid resilience, enabling proliferation even in harsh environments [52,53,54]. Furthermore, *Bacillus* can synthesize lipopeptides, surface proteins, rheumatic acid, fengmycin, and other antibacterial compounds to suppress the proliferation of other bacteria [55]. Therefore, it constituted a significant portion of the living bacterial community in fermented grains. 

The main differential fungal genera were *Saccharomycosis*, *Issatchenkia, Aspergillus* and *Pichia*. *Saccharomycopsis* can generate a diverse array of compounds including esters, lactones, alcohols, acids, and aldehydes during the fermentation process of fermented grains; *Issachenkia*, characterized by its acid and heat resistance traits, predominantly originated from low-temperature Daqu and exhibited proficient alcohol production capabilities [56,57,58]. *Saccharomycosis*, *Issatchenkia* and *Pichia* are all categorized as non-*Saccharomyces* yeasts. It has been reported that *Saccharomyces cerevisiae* is capable of impeding the proliferation of other yeasts through cell–cell interactions during mixed-yeast fermentation [59]. Moreover, in another investigation concerning Baijiu, it was observed that the suppression of *Pichia* growth by *S. cerevisiae* primarily took place during the initial fermentation phase. Subsequently, as fermentation advances, *Pichia* was no longer subject to inhibition and gradually emerged as the dominant genus [60]. In the current study, it was noted that the relative abundance of *Pichia* was greater in the DV15 group in comparison to the DT15 group, while the relative abundance of the other two non-*Saccharomyces* yeast species was diminished. This outcome aligned perfectly with the findings of the aforementioned study, which elucidated the dynamics of yeast interactions during fermentation. The samples utilized in this study were derived from the mid-fermentation stage of fermented grains. At this stage, *Pichia* was no longer under the inhibitory effect of *S. cerevisiae*. However, it was plausible that the growth of *Saccharomycosis* and *Issatchenkia* was suppressed. Consequently, *Pichia* thrived, leading to a proportional increase, while the other two non-*Saccharomyces* yeast species experienced a decline within the living microbial community compared to their relative abundance in the total microbial community. The findings from the DV15 group portrayed the structure of the living microbial community, potentially mirroring the authentic microbial composition during Baijiu fermentation. This outcome further underscored the efficacy of the PMA-amplicon sequencing methodology.

## 5. Conclusions

This study extensively detailed the optimization process of the PMA dye for sample pretreatment in fermented grains and assessed the effectiveness of viable microorganism evaluation. Leveraging PMA pretreatment technology and amplicon sequencing, the researchers successfully devised an efficient method for high-throughput analysis of the living microbial community in Baijiu fermented grains. This method enables the analysis of changes within the living microbial community, thereby offering a novel approach to investigating and enhancing the comprehension of Baijiu fermentation mechanisms. Most conventional fermented foods, akin to Chinese Baijiu, undergo natural fermentation processes, wherein the fermentation cascade encompasses the proliferation, metabolic activities, and ecological succession of a diverse array of microbial consortia. Therefore, this study’s findings offer methodological references for analyzing living microbial communities in such foods. This contribution holds significant importance in deepening our understanding of the fermentation mechanisms underlying traditional fermented foods.

## Data Availability

The original contributions presented in the study are included in the article and Supplementary Materials; further inquiries can be directed to the corresponding author.

## Supplementary Materials

The following supporting information can be downloaded at: https://www.mdpi.com/article/10.3390/foods13111782/s1, Figure S1: Gel images of total bacterial PCR products obtained from samples treated with different PMA concentrations. 1~5: 0, 50, 100, 150, 200 μM PMA treatment; Figure S2: Gel images of total bacterial PCR products obtained from samples treated with different dark incubation treatments. 1~5: 0, 5, 10, 15, 20 min dark incubation treatments; Figure S3: The melting curve and standard curve for quantitative analysis of bacteria and fungi during the fermentation process; Figure S4: The standard curve for quantifying bacteria under different concentrations of PMA treatment; Figure S5: The standard curve for quantifying bacteria under different dark incubation time treatment; Figure S6: The standard curve for quantifying bacteria under different exposure time treatments; Figure S7: Rarefaction curves of amplicon sequencing.
