# Supplementary material for: Selective Elucidation of Living Microbial Communities in Fermented Grains of Chinese Baijiu: Development of a Technique Integrating Propidium Monoazide Probe Pretreatment and Amplicon Sequencing
Source: Foods. 2024 Jun 6;13(11):1782. doi: 10.3390/foods13111782 (PMC11171695; doi:10.3390/foods13111782)
Supplement: Supplementary file 1 [file foods-13-01782-s001.zip › foods-2977532-supplementary.pdf]

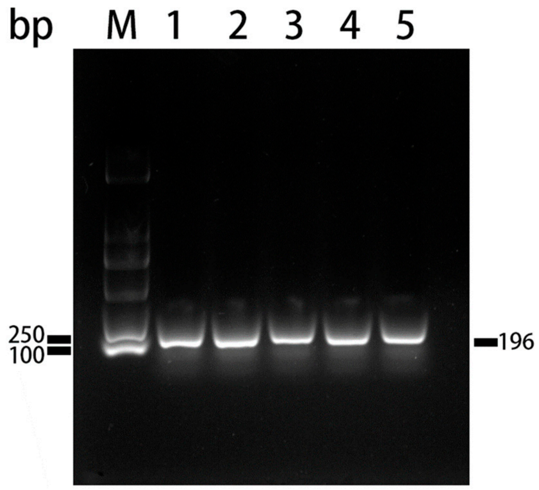

Figure S1 Gel images of total bacterial PCR products obtained from samples treated with different PMA concentrations

1~5: 0, 50, 100, 150, 200 μmol/L PMA treatment

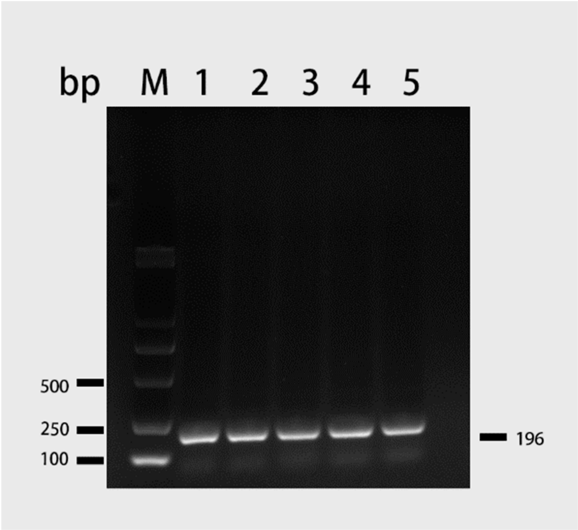

Figure S2 Gel images of total bacterial PCR products obtained from samples treated with different dark incubation treatments

1~5: 0, 5, 10, 15, 20 min dark incubation treatments

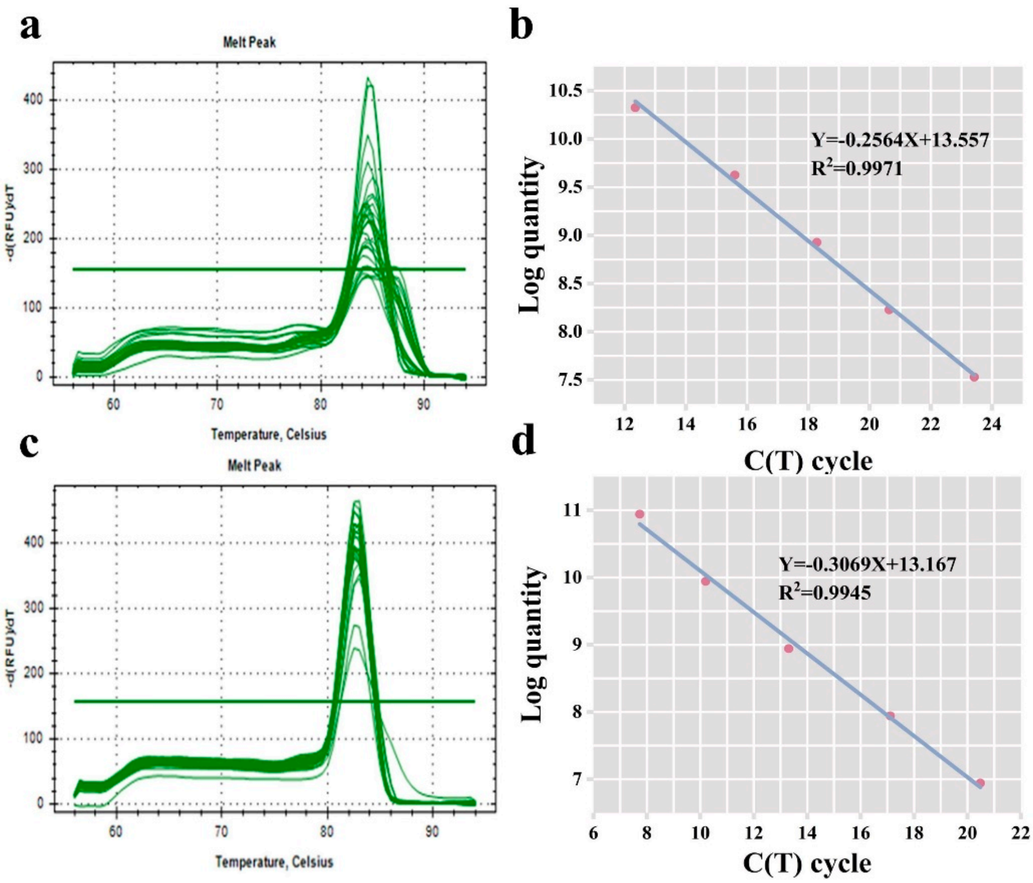

Figure S3The melting curve and standard curve for quantitative analysis of bacteria and fungi during the fermentation process.

a,c. Melting curve analysis of qPCR products of bacteria and fungi; b,d. The standard curve for quantifying bacteria and fungi

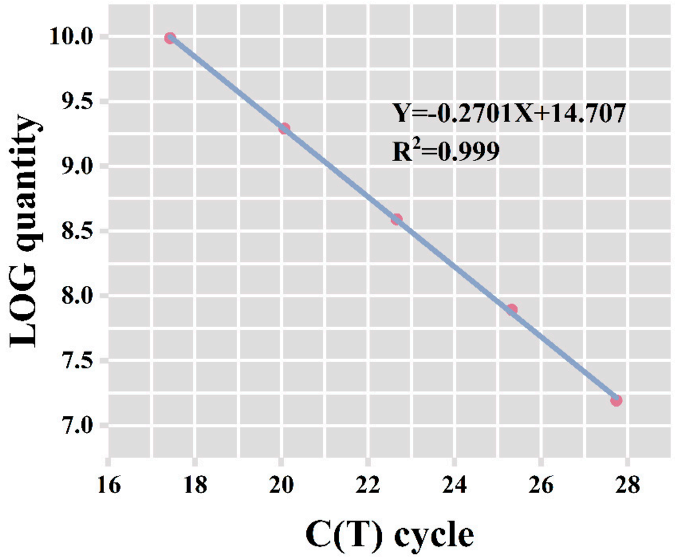

Figure S4 The standard curve for quantifying bacteria under different concentrations of PMA treatment.

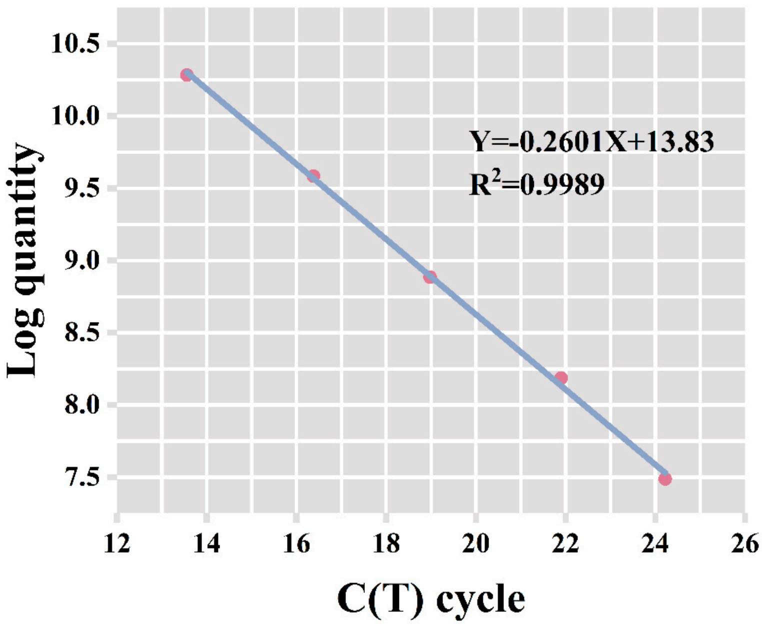

Figure S5 The standard curve for quantifying bacteria under different dark incubation time treatment

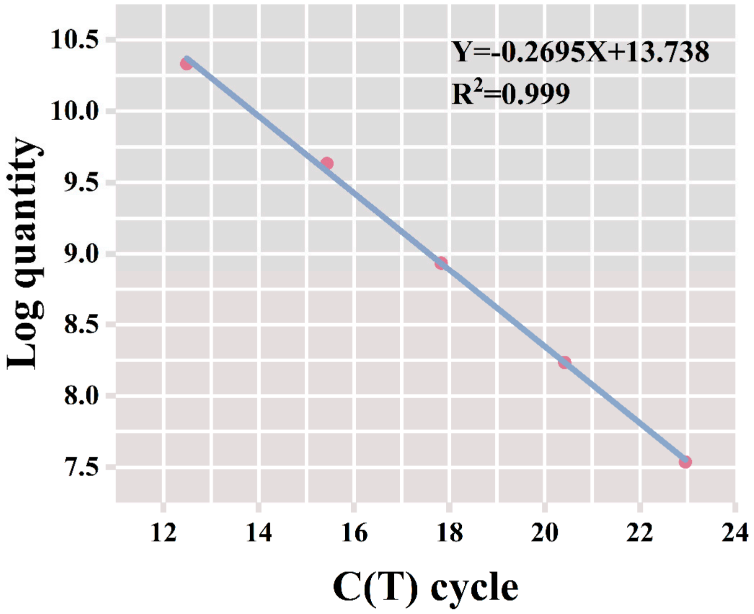

Figure S6 The standard curve for quantifying bacteria under different exposure time treatments.

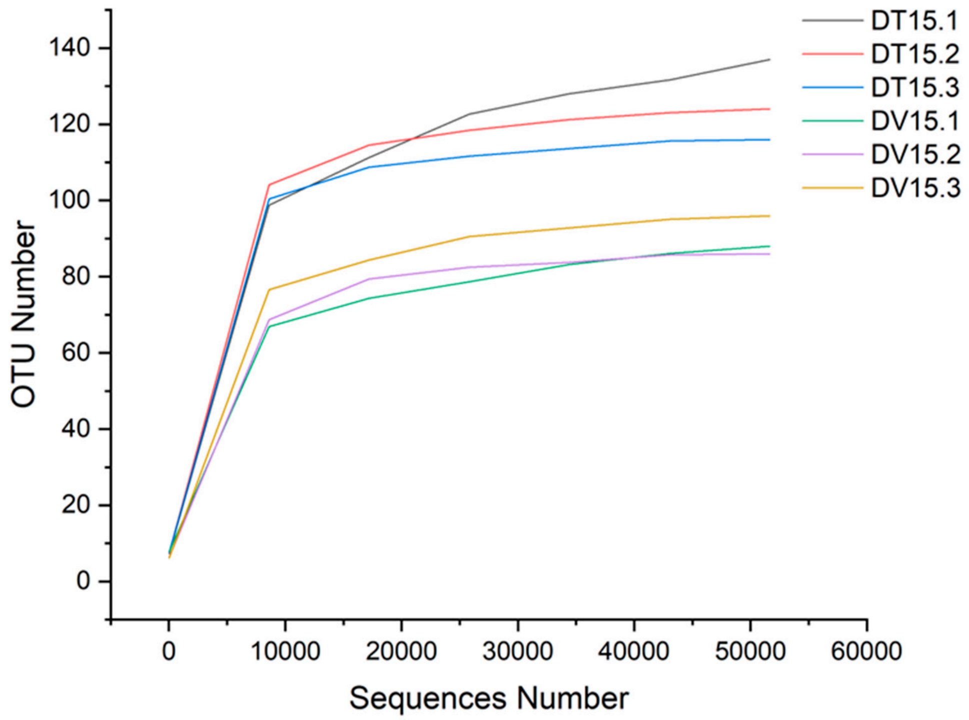

Figure S7 Rarefaction curves of amplicon sequencing
